# Effect of Body Mass Index on Posttonsillectomy Hemorrhage

**DOI:** 10.1155/2017/9610267

**Published:** 2017-05-07

**Authors:** Tetsuro Hoshino, Tohru Tanigawa, Gen Yanohara, Kenta Murotani, Yuichiro Horibe, Toyoaki Murohara, Rei Shibata, Hiromi Ueda

**Affiliations:** ^1^Department of Otolaryngology, Aichi Medical University, Aichi, Japan; ^2^Division of Biostatistics, Clinical Research Center, Aichi Medical University, Aichi, Japan; ^3^Department of Cardiology, Nagoya University Graduate School of Medicine, Nagoya, Japan; ^4^Department of Advanced Cardiovascular Therapeutics, Nagoya University Graduate School of Medicine, Nagoya, Japan

## Abstract

**Objective:**

Obesity affects adverse outcomes in patients undergoing various surgeries. Tonsillectomy is one of the most common surgical procedures and posttonsillectomy hemorrhage (PTH) is the major complication in patients with tonsillectomy. However, the effect of body mass index (BMI) on posttonsillectomy bleeding episodes is not well known. This study aimed to assess the clinical association between obesity and PTH.

**Methods:**

A total of 98 tonsillectomies were retrospectively reviewed. Patient charts were analyzed regarding demographic data and the indication for surgery. Patients with PTH were compared with uneventful cases. Patients were divided into three groups based on BMI: normal weight (BMI < 25 kg/m^2^), overweight (BMI ≥ 25 and <30 kg/m^2^), and obese (≥30 kg/m^2^).

**Results:**

PTH occurred in 13% of patients with normal weight, in 23.5% of patients with overweight, and in 50% patients with obesity. The occurrence of PTH was significantly higher in patients with obesity than in those with normal weight and overweight (*p* = 0.008). Multivariate analysis showed that obesity was a significant factor affecting the incidence of PTH after adjusting for confounding factors.

**Conclusions:**

Our findings suggest that the obese condition is independently associated with the incidence of PTH.

## 1. Introduction

Obesity has become a major health problem in industrial countries with an increasing prevalence in adults and children [1]. Obesity affects adverse outcomes in patients undergoing various types of surgery [2].

Tonsillectomy is one of the most common surgical procedures in the field of otorhinolaryngology. Posttonsillectomy hemorrhage (PTH) is the major complication in patients after tonsillectomy. The overall hemorrhage rate is approximately 10% [3]. Although some potential risk factors for PTH have been investigated [4–14], the effect of body mass index (BMI) on posttonsillectomy bleeding episodes in adults is not well known [15, 16].

In the present study, we investigated the clinical association between obesity and PTH.

## 2. Materials and Methods

### 2.1. Patients

We assessed 98 patients who underwent tonsillectomy at the Department of Otolaryngology, Aichi Medical University, from August 2011 through December 2013. BMI was calculated according to Quetelet (kg/m^2^). Patients were divided into three groups based on BMI: normal weight (BMI < 25 kg/m^2^), overweight (BMI ≥ 25 and <30 kg/m^2^), and obese (≥30 kg/m^2^). This BMI classification system is endorsed by the World Health Organization and the National Institutes of Health. This system is the most widely accepted means of stratifying individuals based on weight [15]. Patients with hematological diseases, deteriorated immune function, chronic wasting disease, and uncontrolled chronic disease were excluded. Children (<12 years old) were excluded because PTH in children is less common than in older patients.

### 2.2. Surgical Methods

Patients underwent surgery under general anesthesia with orotracheal intubation. We inserted a Boyle–Davis mouth gag with the patient in the supine position. The tonsil was removed by blunt peeling with a cotton ball [3]. Hemostasis of the tonsillar fossa was ablated using the Bayonet bipolar device (FC-100A; Daiichi Medical Co., Ltd., Tokyo, Japan). The inferior pole of the tonsil was ablated in all cases and the tonsil was completely removed under direct vision. If surgeons decided that ligation was necessary, ligatures were added using silk. We completed the operation after complete hemostasis of both tonsillar fossae was achieved. Patients were admitted to the hospital 1 to 3 days before surgery. Patients were usually hospitalized for 7 days after surgery. If necessary, the length of hospitalization was extended to more than 8 days after surgery.

### 2.3. Definition of Posttonsillectomy Hemorrhage

We defined PTH as any hemorrhagic episode after extubation. PTH was categorized according to the severity of the hemorrhagic episode as follows: (I) minimal hemorrhage that was controlled after noninvasive treatment, (II) hemorrhage requiring treatment with local anesthesia, and (III) hemorrhage requiring revision surgery under general anesthesia (Table 1) [3]. The period since tonsillectomy to PTH was also recorded.

### 2.4. Statistical Analysis

The effect of 10 factors (BMI, sex, age, indication, tonsil size, platelet count, activated partial thromboplastin time, surgeon's experience, blood loss, and with or without ligation) on PTH was evaluated using Fisher's extract test or the Wilcoxon rank sum test. Variables with *p* < 0.05 in univariate analysis were incorporated into the multivariable model. All statistical analyses were performed using SAS 9.4 (SAS Institute Inc., Cary, NC, USA). A *p* value of <0.05 was considered statistically significant. The study protocol was approved by the Ethics Review Committee of our hospital.

## 3. Results

### 3.1. Baseline Characteristics

**Figure 1:**
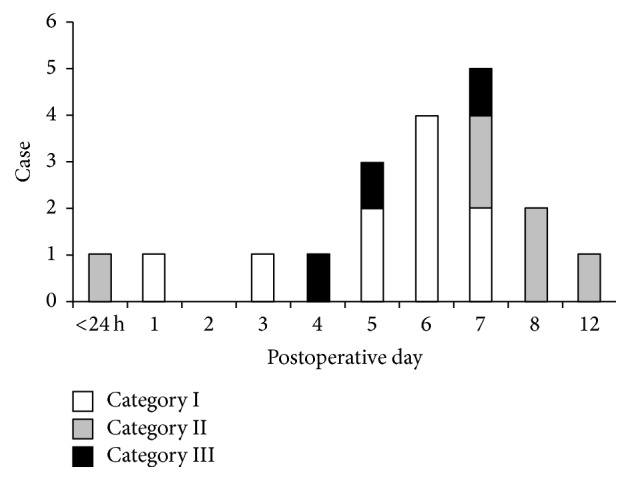
*Posttonsillectomy hemorrhage*: posttonsillectomy hemorrhage (PTH) was observed in 19 (19.4%) patients. Of these 19 patients, one (5.3%) had primary hemorrhage and 18 (94.7%) had secondary hemorrhage. All PTH episodes occurred within 12 days after tonsillectomy. PTH episodes occurred most frequently on postoperative days 7 (5 patients) and 6 (4 patients). Category III PTH (reoperation under general anesthesia) was observed in three patients. Therefore, the overall risk of reoperation was 3.1% (3/98).

PTH occurred in 19 of 98 (19.4%) patients. PTH occurred most frequently on postoperative days 6 and 7 (4 and 5 patients, resp.). Category III PTH (reoperation under general anesthesia) was observed in three (3.1%) patients. All cases of PTH occurred within 12 days after tonsillectomy (Figure 1).

The occurrence rate of PTH in male patients was significantly higher than that in female patients (Table 2, *p* = 0.040). The mean age in patients with or without PTH was 33.4 years and 32.6 years, respectively. Age did not affect the occurrence of PTH. PTH was observed in none of 55 (16.4%) patients with chronic tonsillitis, two of 21 (7.7%) patients with IgA nephropathy, and eight of 23 (42.1%) patients with sleep apnea syndrome (SAS). The occurrence of PTH was significantly higher in patients with SAS, but not in those with chronic tonsillitis and IgA nephropathy (*p* = 0.040). There were no significant differences in platelet count and activated partial thromboplastin time between patients with or without PTH. There were also no significant differences in the surgeon's experience, with or without ligation, and total blood loss between patients with or without PTH.

Normal weight was observed in 69 (70.4%) patients, overweight in 17 (17.3%), and obesity in 12 (12.2%). PTH occurred in nine of 69 (13%) patients with normal weight, in four of 17 (23.5%) patients with overweight, and in six of 12 (50%) patients with obesity. The occurrence rate of PTH was significantly higher in patients with obesity than in those with normal weight and overweight (Table 2, *p* = 0.012).

### 3.2. Effects of Various Factors on Posttonsillectomy Hemorrhage

To determine the factors associated with the occurrence of PTH, we performed multiple logistic regression analyses. Variables with *p* < 0.05 in univariate analysis were entered into multivariate logistic regression with stepwise selection. This variable selection procedure retained obstructive SAS and BMI in the final model.  Table 3 shows the results of multivariate logistic regression with stepwise selection. Obesity was a significant factor that affected the incidence of PTH after adjusting for sex and etiology of obstructive SAS (odds ratio, 4.55; 95% confidence interval, 1.10–18.8; *p* = 0.036; Table 3).

## 4. Discussion

This study demonstrated, for the first time, that the obese condition was significantly associated with posttonsillectomy bleeding episodes in adolescents and adults. Our data suggest that patients with obesity who undergo tonsillectomy should be carefully followed up after the operation. Our findings have several implications for informed consent of patients who intend to undergo tonsillectomy. If patients are in the state of obesity before tonsillectomy, patients should reduce weight until surgery. Surgeons should be aware of frequent hemorrhage in obese patients and prepare for sudden bleeding.

Obesity affects wound healing, wound infection rate, and bleeding risk following various interventions [2, 17]. In addition, there are concerns about patients with obesity regarding the technical difficulty in performing tonsillectomy [18]. Obesity disturbs hemostasis because a wide operating field cannot be obtained because of abundant visceral fat. Therefore, wound issues and technical difficulty might cause a high rate of occurrence of PTH in patients with obesity.

Our study showed that obesity was significantly associated with posttonsillectomy bleeding episodes. In contrast to this finding, a previous report showed that overweight or obesity did not increase the risk for PTH [15]. The reason for this discrepancy between studies is unclear. A possible reason for the difference between studies is the difference in criteria for patients' inclusion. Our study was only conducted in Japanese patients who were 12 years or older. Another possible reason is due to the difference of indications for tonsillectomy. We had undergone elective tonsillectomy in all cases, whereas Riechelmann et al. performed abscess tonsillectomy in 65 of 300 patients (21.7%). The risk of bleeding following abscess tonsillectomy seems higher than that in elective tonsillectomy [19].

In conclusion, our findings suggest that the obese condition in adolescents and adults is independently associated with the incidence of PTH. Therefore, nutritional approaches aimed at reducing body weight before tonsillectomy could be useful for an uncomplicated postoperative course.

## Figures and Tables

**Table 1:** Classification of posttonsillectomy hemorrhage (PTH).

Category I	Minimal hemorrhage, controlled after noninvasive treatment
Category II	Hemorrhage requiring treatment with local anesthesia
Category III	Revision surgery under general anesthesia

**Table 2:** Comparison of patients' characteristics between the PTH and non-PTH groups.

	Total *N* = 98	PTH (%) *N* = 19	Non-PTH (%) *N* = 79	*p* value
Sex				0.040
Male	56	15 (26.8)	41 (73.2)	
Female	42	4 (9.5)	38 (90.5)	
Age (y)	32.7 ± 13.5	33.4 ± 16.6	32.6 ± 12.7	0.986
Indication				0.040
Chronic tonsillitis	55	9 (16.4)	46 (83.6)	
IgA nephropathy	23	2 (8.7)	21 (91.3)	
OSAS	19	8 (42.1)	11 (57.9)	
Benign tumor suspected	1	0	1 (100)	
Tonsil size				0.444
I	51	8 (15.7)	43 (84.3)	
II or III	47	11 (23.4)	36 (76.6)	
Platelet count (×10^4^)	25.4 ± 5.3	25.2 ± 3.7	25.5 ± 5.6	0.857
APTT	28.5 ± 2.8	29.3 ± 3.3	28.4 ± 2.7	0.207
Surgeon's experience (y)	2.0 ± 1.2	1.8 ± 1.2	2.1 ± 1.2	0.520
Blood loss (mL)	12.3 ± 20.3	10.1 ± 13.6	12.8 ± 21.6	0.692
Ligation				1.000
With	57	11 (19.3)	46 (80.7)	
Without	41	8 (19.5)	33 (80.5)	
BMI (kg/m^2^)				0.012
BMI < 25	69	9 (13.0)	60 (87.0)	
BMI ≥ 25 and <30	17	4 (23.5)	13 (76.5)	
BMI ≥ 30	12	6 (50)	6 (50)	

PTH: posttonsillectomy hemorrhage, IgA: immunoglobulin A, OSAS: obstructive sleep apnea syndrome, APTT: activated partial thromboplastin time, and BMI: body mass index.

**Table 3:** Results of multivariate logistic regression with stepwise selection.

Variable	Category	OR	95% CI	*p* value
OSAS		3.09	0.93–10.3	0.066
BMI	BMI < 25	1		
	BMI ≥ 25 and <30	1.65	0.42–6.54	0.474
	BMI ≥ 30	4.55	1.10–18.8	0.036

## References

[1] Shibata R., Ouchi N., Takahashi R. (2012). Omentin as a novel biomarker of metabolic risk factors. *Diabetology and Metabolic Syndrome*.

[2] Birkmeyer N. J. O., Charlesworth D. C., Hernandez F. (1998). Obesity and risk of adverse outcomes associated with coronary artery bypass surgery. *Circulation*.

[3] Ikoma R., Sakane S., Niwa K., Kanetaka S., Kawano T., Oridate N. (2014). Risk factors for post-tonsillectomy hemorrhage. *Auris Nasus Larynx*.

[4] Arnoldner C., Grasl M. C., Thurnher D. (2008). Surgical revision of hemorrhage in 8388 patients after cold-steel adenotonsillectomies. *Wiener Klinische Wochenschrift*.

[5] Kim M. K., Lee J. W., Kim M. G., Ha S. Y., Lee J. S., Yeo S. G. (2012). Analysis of prognostic factors for postoperative bleeding after tonsillectomy. *European Archives of Oto-Rhino-Laryngology*.

[6] Kværner K. J. (2009). Benchmarking surgery: secondary post-tonsillectomy hemorrhage 1999–2005. *Acta Oto-Laryngologica*.

[7] Perkins J. N., Liang C., Gao D., Shultz L., Friedman N. R. (2012). Risk of post-tonsillectomy hemorrhage by clinical diagnosis. *The Laryngoscope*.

[8] Sarny S., Habermann W., Ossimitz G., Stammberger H. (2012). Significant post-tonsillectomy pain is associated with increased risk of hemorrhage. *Annals of Otology, Rhinology and Laryngology*.

[9] Sarny S., Ossimitz G., Habermann W., Stammberger H. (2011). Hemorrhage following tonsil surgery: a multicenter prospective study. *Laryngoscope*.

[10] Sarny S., Habermann W., Ossimitz G., Schmid C., Stammberger H. (2011). Tonsilar haemorrhage and re-admission: a questionnaire based study. *European Archives of Oto-Rhino-Laryngology*.

[11] Sarny S., Ossimitz G., Habermann W., Stammberger H. (2013). Preoperative coagulation screening prior to tonsillectomy in adults: current practice and recommendations. *European Archives of Oto-Rhino-Laryngology*.

[12] Tami T. A., Parker G. S., Taylor R. E. (1987). Post-tonsillectomy bleeding: an evaluation of risk factors. *Laryngoscope*.

[13] Tolska H. K., Takala A., Pitkäniemi J., Jero J. (2013). Post-tonsillectomy haemorrhage more common than previously described-an institutional chart review. *Acta Oto-Laryngologica*.

[14] Tomkinson A., Harrison W., Owens D., Harris S., McClure V., Temple M. (2011). Risk factors for postoperative hemorrhage following tonsillectomy. *Laryngoscope*.

[15] Riechelmann H., Blassnigg E. C., Profanter C., Greier K., Kral F., Bender B. (2014). No association between obesity and post-tonsillectomy haemorrhage. *Journal of Laryngology and Otology*.

[16] Schrock A., Send T., Heukamp L., Gerstner A. O., Bootz F., Jakob M. (2009). The role of histology and other risk factors for post-tonsillectomy haemorrhage. *European Archives of Oto-Rhino-Laryngology*.

[17] Doyle S. L., Lysaght J., Reynolds J. V. (2010). Obesity and post-operative complications in patients undergoing non-bariatric surgery. *Obesity Reviews*.

[18] Ishida R., Nakamura S., Fuke T., Yamada H. (2006). Post-tonsillectomy hemorrhage: a retrospective study of 503 operations. *Practica Oto-Rhino-Laryngologica*.

[19] Giger R., Landis B. N., Dulguerov P. (2005). Hemorrhage risk after quinsy tonsillectomy. *Otolaryngology—Head and Neck Surgery*.

